# Dynamics of carbon sequestration in vegetation affected by large-scale surface coal mining and subsequent restoration

**DOI:** 10.1038/s41598-024-64381-1

**Published:** 2024-06-12

**Authors:** Yaling Xu, Jun Li, Chengye Zhang, Simit Raval, Li Guo, Fei Yang

**Affiliations:** 1 College of Geoscience and Surveying Engineering, China University of Mining and Technology-Beijing, Beijing, 100083 China; 2 School of Minerals and Energy Resources Engineering, University of New South Wales, Sydney, 2052 Australia; 3 State Key Laboratory of Coal Resources and Safe Mining, China University of Mining and Technology-Beijing, Beijing, 100083 China

**Keywords:** Environmental sciences, Environmental impact

## Abstract

Surface coal development activities include mining and ecological restoration, which significantly impact regional carbon sinks. Quantifying the dynamic impacts on carbon sequestration in vegetation (VCS) during coal development activities has been challenging. Here, we provided a novel approach to assess the dynamics of VCS affected by large-scale surface coal mining and subsequent restoration. This approach effectively overcomes the limitations imposed by the lack of finer scale and long-time series data through scale transformation. We found that mining activities directly decreased VCS by 384.63 Gg CO_2_, while restoration activities directly increased 192.51 Gg CO_2_ between 2001 and 2022. As of 2022, the deficit in VCS at the mining areas still had 1966.7 Gg CO_2_. The study highlights that complete restoration requires compensating not only for the loss in the year of destruction but also for the ongoing accumulation of losses throughout the mining lifecycle. The findings deepen insights into the intricate relationship between coal resource development and ecological environmental protection.

## Introduction

The rapid growth of global economy has led to an increase in energy demand^1^. As one of foremost fossil fuels, coal has witnessed a historic milestone in 2023, surpassing a staggering 8.5 billion tons in global consumption for the first time (source: International Energy Agency, IEA). However, coal mining, especially surface coal mining, has significantly disturbed the natural vegetation and soils. This destructive practice not only leads to large areas of land degradation, but also has a severe impact on the regional carbon balance^2,3^. To achieve the targets for absolute carbon reduction set by the United Nations Framework Convention on Climate Change (UNFCCC) and the Paris Agreement, it is essential to consistently enhance the carbon sequestration capacity of ecosystems in mining areas and implement significant ecological protection and restoration projects^4–6^. Vegetation plays a crucial role as a carbon sink in the carbon cycle of terrestrial ecosystems^7–9^. Its carbon sequestration capacity is a core objective of ecological protection and restoration in mining areas^10–12^. Therefore, quantifying the impacts of surface coal mining and restoration activities on carbon sequestration in vegetation (VCS) can provide essential data to achieve ecological balance in the coal industry^13,14^.

The carbon sinks of vegetation in surface coal mining areas typically undergo three stages of “natural vegetation-mining-restoration”. Surface mining completely removes vegetation and soils, resulting in the conversion of the original vegetation into carbon source sites such as mine pits or industrial sites^15^. The destroyed area completely loses the VCS capacity compared to the previous year, resulting in a direct change in VCS. This change is evident and directly related to the mining activities. During the mining process, the destroyed area remains devoid of vegetation and lacks the VCS capacity. Hence, carbon that should have been sequestered is lost, known as the potential loss of VCS. During the restoration process, the destroyed area is replanted with vegetation, allowing it to regain its VCS capacity.

Many scholars have investigated carbon sinks in different stages of mining areas. However, most studies have focused on changes in vegetation carbon stocks (static carbon)^16–21^, with few examining changes in the VCS capacity (dynamic carbon). Current remote sensing methods to analyze the impacts of mining and restoration activities on VCS typically involve two steps. Firstly, the study area is selected, and an appropriate method for calculating VCS is chosen based on the area's characteristics. Secondly, statistical methods are used to analyze the spatio-temporal characteristics of VCS. Current methods to calculate carbon sinks using remote sensing focus on forests^22–25^, grasslands, and similar environments. These methods usually have low spatial resolution, which does not meet the requirements for long-term and finer scale data in large-scale mining areas^26,27^. Existing studies on spatio-temporal changes in VCS can be classified into two categories. The first category primarily employs temporal data to analyze the trends of VCS within large regions, which only captures the macro-level spatio-temporal variability of VCS changes within the study area^28–31^. Although comprehensive surveys are valuable in assessing VCS, they may overlook the dynamics of what is occurring in the destroyed and restored areas. Furthermore, it is important to note that climate significantly influences inter-annual changes in VCS^32,33^. Therefore, direct statistics on regional variations in the VCS do not fully reflect the impact of mining, making it difficult to measure the differences in the VCS at different stages of production in open-pit mines. A more detailed analysis is necessary to achieve a more comprehensive understanding of VCS changes. The second category utilize data on land use classification in mining areas to investigate the impact of various land use changes on VCS^34–38^. The emphasis is on capturing changes in VCS in destruction and restoration areas. Nevertheless, existing studies often face the limitation of low temporal frequency, usually occurring only once every five years, due to the lack of comprehensive and detailed data on land use classification. Consequently, capturing the dynamic changes in carbon sinks within mining areas become challenging.

In summary, there is a shortage of methods for calculating long-term and finer scale carbon sink data that are applicable to mining scenarios that are widespread but single mines are small in scope. Additionally, there is a lack of monitoring data on mining activities. This means that when dealing with large-scale mining areas, existing remote sensing methods can only calculate changes in carbon sinks at a macro level, which does not directly reflect the impact of mining and rehabilitation activities on carbon sinks. Furthermore, previous studies have not considered the potential changes of VCS due to surface mining. Therefore, there is an urgent needed for a method to quantify the direct and potential impacts of coal mining and restoration activities on VCS. This will clarify the pattern of impacts on VCS during the entire cycle of coal development.

To address the abovementioned challenges, in this work, a new approach has been provided to assess the dynamic impact from mining and restoration activities on VCS in large-scale surface coal mining areas. This approach effectively overcoming the limitations imposed by the lack of finer scale data and long time series data through scale transformation. The analysis was focused on the dynamic change of VCS in the areas of vegetation destruction and restoration based on the spatio-temporal data on vegetation disturbance. This successfully quantified the direct and potential changes from mining and restoration activities. Furthermore, the concept of the deficit in carbon sequestration in vegetation (VCSD) was introduced. This study compared 133 open-pit mines in the Shendong coal base using the indicators “VCSD” and “potential changes in VCS per unit area of restoration” to reveal variations in carbon sequestration and sinks among the mines. These findings contribute to the analysis of the impact of coal mine development activities on the local carbon cycle and the calculation of carbon sink compensation.

## Results

### Spatio-temporal variation in VCS

**Figure 1:**
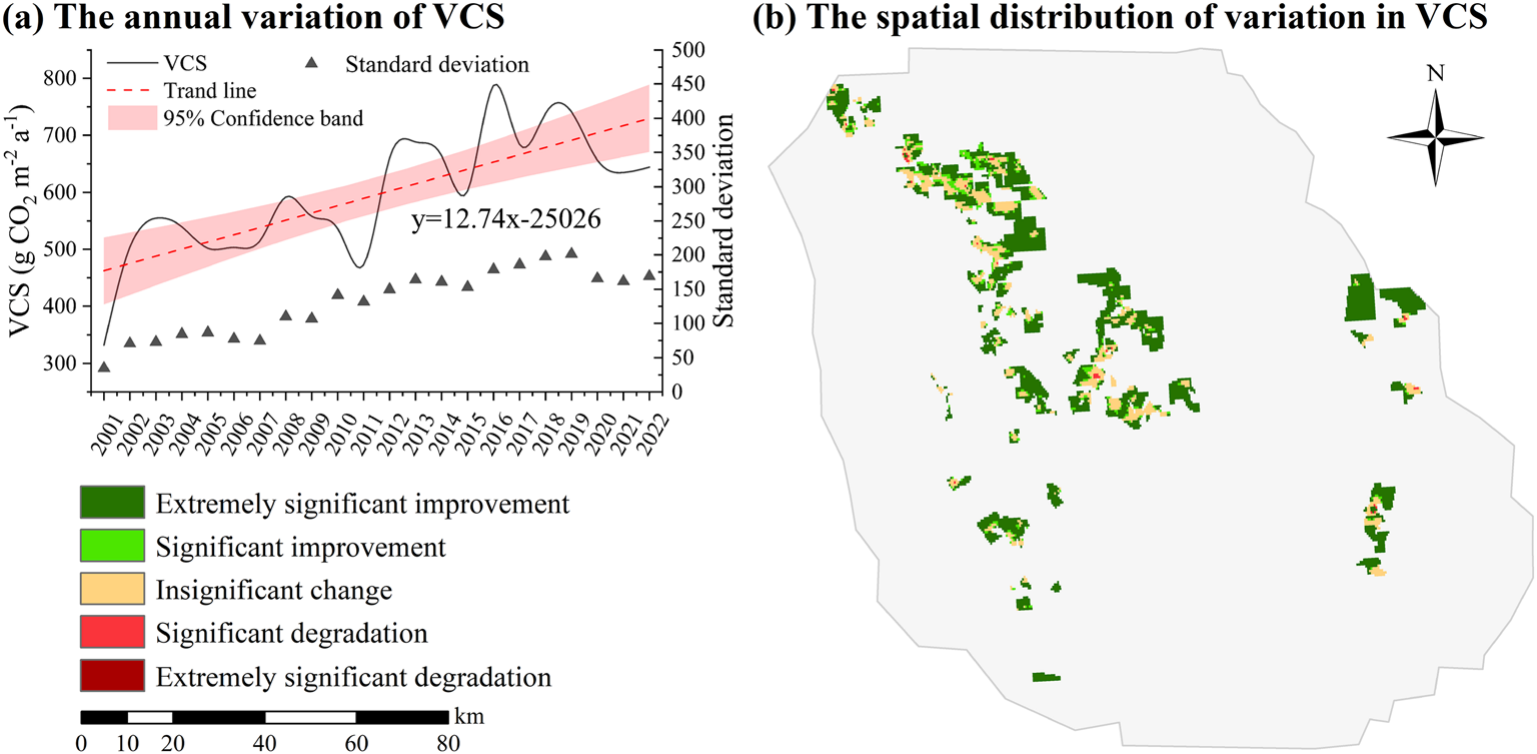
The VCS in the study areas from 2001 to 2022. (**a**) The annual variation of VCS; (**b**) The spatial distribution of variation in VCS. The changes in VCS were determined using a least-squares linear regression model, which provided the slope representing the trends.

There was interannual variation in VCS within the 133 open-pit mines (Fig. 1a). Over the 20 years, the annual VCS ranged from 331.94 to 787.32 g CO_2_ m^−2^ a^−1^, with an average of 596.04 g CO_2_ m^−2^ a^−1^. The trend analysis revealed a gradual increase in the annual VCS, with a 12.74 g CO_2_ m^−2^ a^−1^ rate. Additionally, the standard deviation of VCS was calculated annually to determine the degree of dispersion. The increasing trend of the standard deviation indicates a strengthening polarization of the annual VCS.

The analysis of the spatial distribution of variation in VCS, as depicted in Fig. 1b, indicated that most areas experienced “Extremely significant improvement” or “Significant improvement” except for the coal mining regions where the changes were “Insignificant change”. This suggested that mining activities had significantly suppressed the positive trend of regional vegetation growth. The overall VCS in the region was influenced by natural climate factors, which exhibited a consistent annual increase without any disturbances. Therefore, focusing solely on changes in VCS in the study area does not adequately reflect the impact of mining activities on vegetation. conducting a more detailed analysis to understand the specific impact of mining on VCS in areas experiencing vegetation disturbances is crucial.

### The impacts of mining and restoration activities on VCS

**Figure 2:**
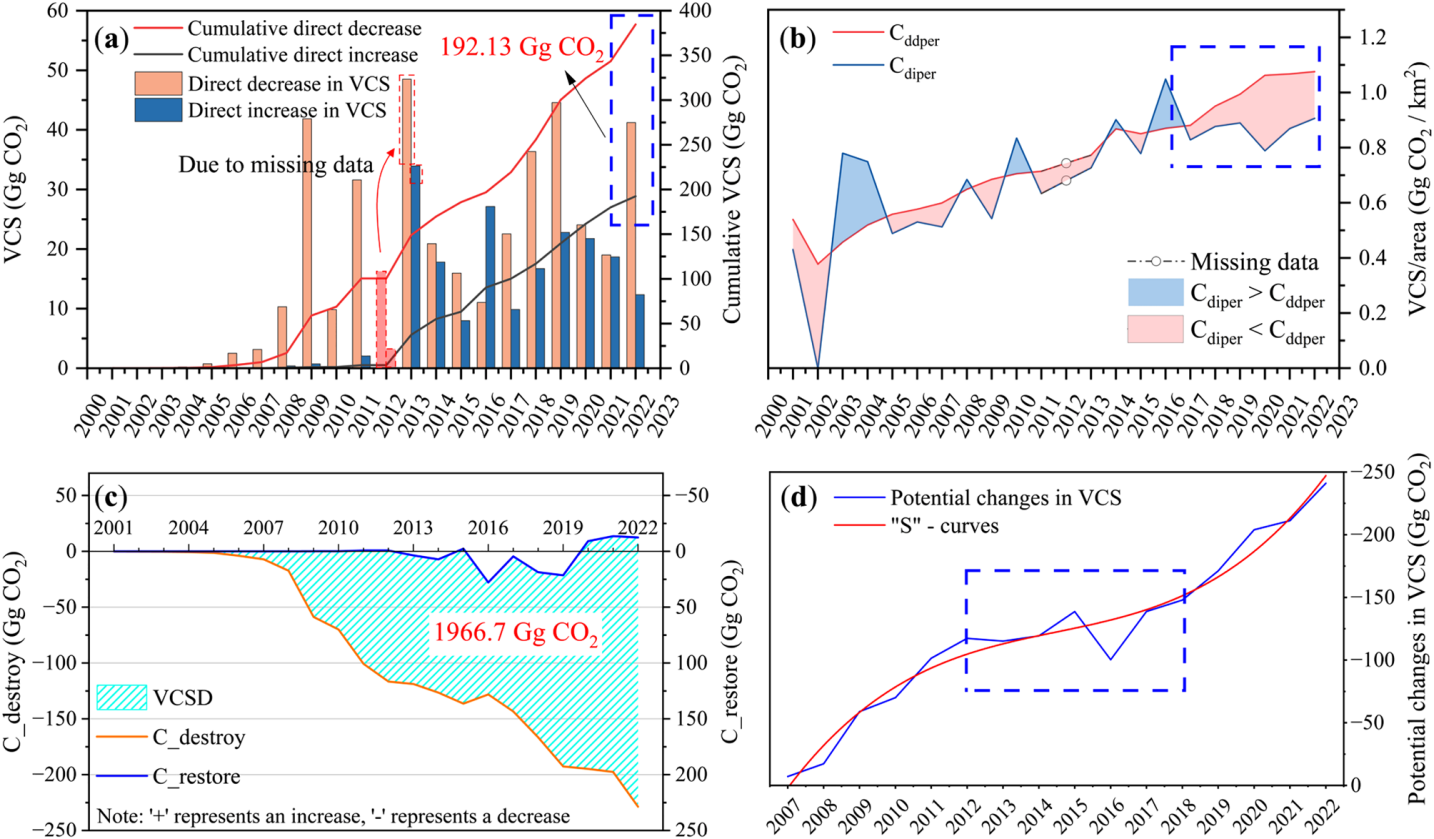
Inter-annual variation in the impacts of mining and restoration activities on VCS in the study area from 2001–2022. (**a**) Direct changes in VCS. As there is no data available for 2012, any disturbance to vegetation that occurred in 2012 will be categorized as occurring in 2013. Therefore, the loss of vegetation carbon sequestration that occurred in that year will be added to the total for 2013, as shown in the dotted box. (**b**) Direct changes in VCS per unit area. The blue box highlights the timeframe during which C_diper_ consistently exceeds C_ddper_. (**c**) The potential changes in VCS of both destroyed and restored areas; (**d**) The potential changes in VCS for each year after 2007.

This paper shifts the research focus to the areas of vegetation destruction and restoration. The impact of mining and rehabilitation activities on the vegetation of the study area were analyzed from two perspectives. The term “direct changes in VCS” refers to changes relative to the previous year when the destruction or restoration occurred, including direct decrease and direct increase (see Materials and Methods). This change is observed in the region of mining and restoration activities that occur annually. The inter-annual variation of direct changes in VCS is shown in Fig. 2a. As illustrated in Supplementary Table S1, the direct change in VCS is proportional to the mining and restoration activities carried out each year. From 2001 to 2022, the direct decrease in VCS amounted to 384.63 Gg CO_2_ (1 Gg = 10^9^ g), while the direct increase in VCS amounted to 192.51 Gg CO_2_. As of 2022, there was a VCS difference of 192.13 Gg CO_2_ between mining and restoration activities. It is worth noting that in 2016, the direct increase was 27.14 Gg CO_2_, which was 2.46 times higher than the direct decrease.

Furthermore, Fig. 2b compares the direct decrease in VCS per unit of destroyed area (C_ddper_) with the direct increase in VCS per unit of restored area (C_diper_). After removing the effect of area on the direct change in VCS, C_ddper_ reflects the level of VCS prior to vegetation destruction, which fluctuates annually in accordance with climate. C_diper_ represents the level of VCS following vegetation restoration, with inter-annual fluctuations influenced by the combined effects of climate and restoration activities. Both C_ddper_ and C_diper_ exhibited an upward trend over time. Before 2017, C_ddper_ and C_diper_ fluctuated with little difference, indicating that the VCS in the restoration area was able to approach the level prior to destruction. However, after 2017, C_ddper_ consistently exceeded C_diper_*,* suggesting that the negative impacts of mining activities on local carbon sinks had outweighed the positive impacts of restoration activities. Supplementary Table S1 counts the annual restoration rate of vegetation (R_V_) and restoration rate of VCS (R_VCS_) in the study area (see Materials and Methods). Both the R_V_ and the R_VCS_ were consistently low before 2011, remaining below 0.1 for most years. From 2013 to 2016, both rates had similar values, with their maximum values reached in 2016 at 2.04 and 2.46, respectively. Overall, the R_VCS_ was equal to R_V_, with both having a value of 0.5. However, since 2017, the R_VCS_ had consistently been lower than the R_V_. Additionally, C_ddper_ had consistently been greater than C_diper_, suggesting that the negative impacts of mining activities on local carbon sinks had outweighed the positive impacts of restoration activities.

Figure 2c illustrates the potential changes in VCS of both destroyed (C_destroy) and restored areas (C_restore) (see Materials and Methods). As mining activities persisted, the C_destroy consistently increased from 2001 to 2022. In contrast, the C_restore fluctuated ranging from -13.57 to 27.79 Gg CO_2_, indicating the success of restoration activities in maintaining the VCS close to the original state. From 2012 to 2019, C_restore exceeded 0, indicating that the vegetation in the restored area sequestered more carbon than its original state. The results indicate that the restoration was effective and that the restoration method was appropriate. As of 2022, the deficit in VCS (VCSD) at the mining areas still had 1966.7 Gg CO_2_ within the 133 open-pit mines in the Shendong coal base, requiring compensation from other sources by local companies. Large-scale mining in the Shendong coal base began in 2007. Figure 2d shows the potential changes in VCS for each year after 2007. Overall, the data reveals a decreasing trend in VCS, which follows an “S” curve over time. Between 2007 and 2012, there was a rapid increase, followed by a brief buffer that coincided with the trough period of the coal industry between 2012 and 2017.

### Spatial distribution in the impact of mining and restoration activities on VCS

**Figure 3:**
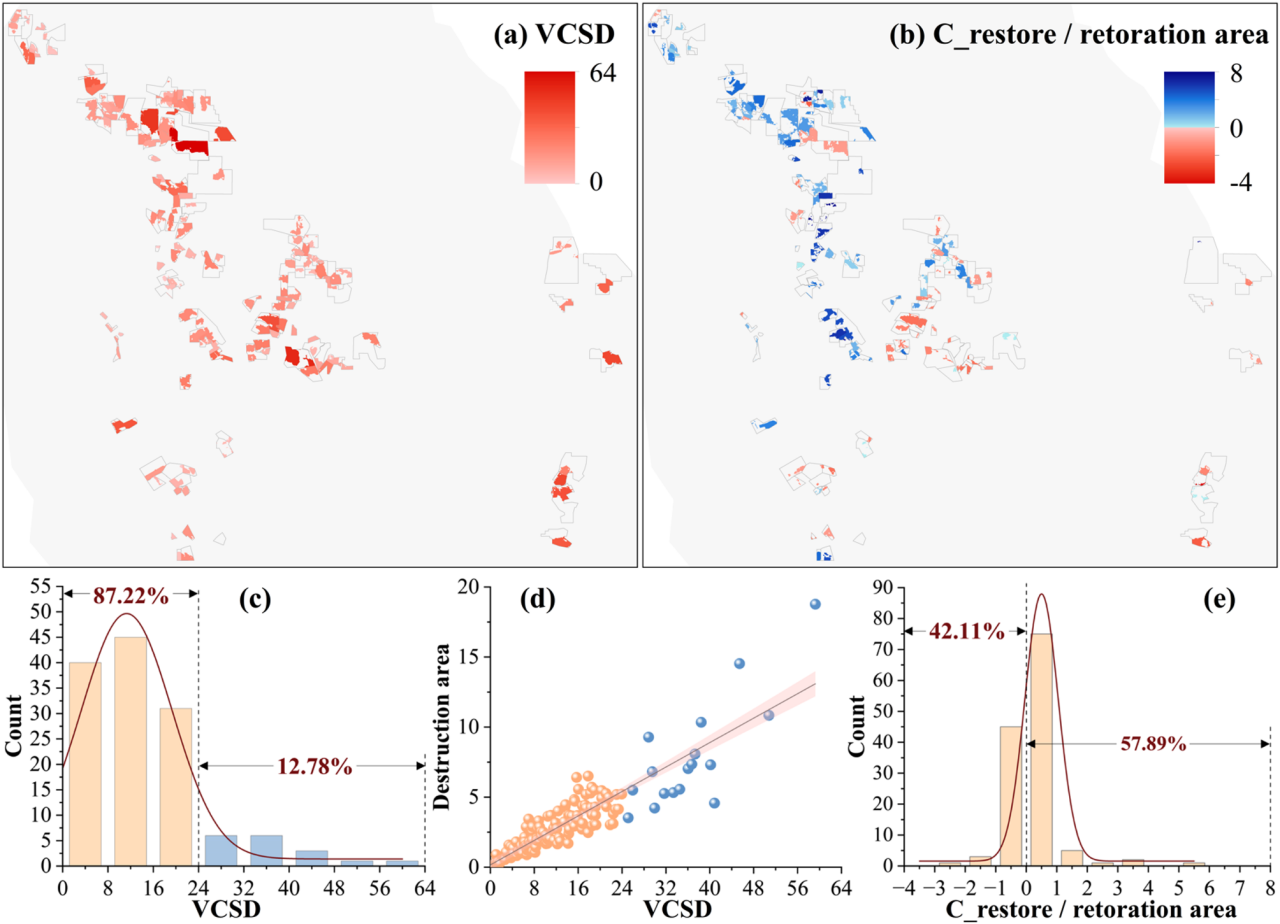
Comparative statistics of 133 open-pit mines. (**a**) The spatial distribution of VCSD for 133 open-pit mines. (**b**) The potential changes in VCS per unit area of restoration. Blue indicates that C_restore is greater than 0, meaning that the vegetation in the restored area has sequestered more carbon than the original level; Red indicates that C_restore is less than 0, meaning that the vegetation in the restored area has sequestered less carbon than the original level. (**c**) The grading statistics of VCSD for 133 open-pit mines. (**d**) The relationship between VCSD and vegetation destruction area. Yellow corresponds to 87.22% of the mines in (**c**), and blue corresponds to 12.78% of the mines. (**e**) The grading statistics of potential changes in VCS per unit area of restoration for 133 open-pit mines.

The VCSD of the study area was quantified at the scale of individual mines, and the spatial distribution for 133 open-pit mines is presented in Fig. 3a. Overall, the VCSD did not vary significantly among the mines. The grading statistics in Fig. 3c reveal that 116 mines had a VCSD between 0 and 24 Gg CO_2_, accounting for 87.22% of the total. There were only a few exceptions that exceeded 24 Gg CO_2_, accounting for 12.78% of the total. Figure 3d illustrates a positive correlation between the VCSD of each mine and its corresponding vegetation destruction area. Most mines with a VCSD of over 24 Gg CO_2_ have a destruction area of up to 5 km^2^.

In order to compare the effectiveness of restoration activities at each mine, Fig. 3b illustrates the potential changes in VCS per unit area of restoration. The spatial distribution indicated a demarcation trend in the southwest-northeast direction. Specifically, most of the mines in the northwest direction exhibited positive changes in VCS. Out of 133 mines, 77 mines (57.89%) had a C_restore greater than 0 per unit of restored area, indicating better restoration effects. Conversely, most mines in the southeast direction exhibited negative changes in VCS, suggesting that the restored vegetation failed to reach its original state. However, according to the grading statistics (Fig. 3e), 95.49% of the mines showed potential changes in VCS of -1 ~ 1 Gg CO_2_ in restored areas. The VCS of most mines were able to approach their original state after the restoration activities, suggesting that restoration activities were effective in mitigating carbon sequestration losses.

## Discussion

### Availability assessment of data

Calculating vegetation NPP typically requires input data such as vegetation indices, meteorological data (including temperature, precipitation, and solar radiation), land cover data, and soil properties^39^. These data are generally collected through satellite remote sensing, meteorological stations, and soil surveys^40,41^. Nevertheless, due to the exorbitant cost of data acquisition and the potential for missing remote sensing data, obtaining long time series and high-frequency NPP data over a wide range of mining areas is challenging. Therefore, for this study, we selected the MODIS product from the available datasets^42^. The MOD17A3 dataset has been widely utilized for vegetation growth, biomass estimation, environmental monitoring, and global change studies at regional or global scales^43^. Studies have shown that the MOD17A3 data product accurately represents the true global NPP^44,45^. The accuracy and validity of this data were assessed by comparing it with similar studies. The NPP for both grass and shrubs fell within acceptable ranges (Table 2). This indicates that the NPP used in this study was feasible.

**Table 2:** Comparison of MOD17A3 NPP and other estimated value.

Method	NPP for grass and shrub (g C m^−2^ a^−1^)	Time	Region	Reference
MOD17A3	76.67—318.56	2001–2022	Shendong coal base	\
CASA	286.93	2010–2015	Changhe Basin mining area	^46^
GLO–PEM	114.76—394.05	2000–2015	Yellow River Basin	^47^
CASA	90—511.00	2006–2020	Shengli mining area	^27^

This study utilized two datasets: spatio-temporal data on vegetation disturbance and NPP data. The vegetation disturbance data was obtained from Auto-VDR, which characterizes the growth status of vegetation using the maximum NDVI of the growing season (July–September). If the NDVI falls below the vegetated/bare ground threshold (*α*) set by Auto-VDR, it can be inferred that the area has been transformed from vegetation to non-vegetation types, such as open-pit or outer dumping sites. As a result, the NPP is 0 due to the absence of VCS capacity. The NPP data, however, was generated using the Biome-BGC model, which considers the land cover type, daily leaf area index (LAI), and daily meteorological data (PAR, precipitation, minimum and maximum temperature, and water vapor pressure deficit) as inputs. The annual NPP used in this study was calculated by summing up the observed data every eight days within a given year. It should be noted that an area identified as destroyed by Auto-VDR may not have been utterly devoid of vegetation throughout the year. Instead, it was only detected as devoid of vegetation during the growing season (July–September). Therefore, it is possible that the destroyed area could still have been vegetated and able to fix carbon in the period before the occurrence of the destruction, resulting in a lower annual NPP. For example, if mining destroys vegetation in June, the maximum NDVI between July and September will be less than *α*, and the area will be identified as destroyed. However, from January to June, vegetation in the area can still sequester carbon, and NPP accumulates. Hence, it is reasonable to expect that MOD17A3 would still produce a lower annual NPP in areas determined as unvegetated by Auto-VDR. This lower value is referred to as NPP_bg_. Therefore, when calculating NPP for areas covered by vegetation at a 30 m resolution, the contribution of this background value must be subtracted from the total NPP of a 500 × 500 m area. The scale transformation allows for the application of higher resolution (500 m) VCS data to a finer scale (30 m) of the mine. This transformation overcomes the limitations imposed by the lack of finer scale NPP data on long time series. This research method can be applied to other types of anthropogenic activities such as deforestation and land use changes. It provides ideas for ecosystem management on a more expansive scale. This study was limited to a 30 m resolution as Landsat is considered the most suitable remote sensing data that is freely available for long time series. Despite the limitations in data accuracy, it is still sufficient for vegetation analysis in this area. This is because the original and restored vegetation in Shendong coal base is relatively homogeneous at a 30 × 30 m scale on the grand (see Supplementary Fig. S1 online). At this resolution, the overall vegetation cover can be effectively captured and analyzed for broad trends in vegetation change within the study area. Consequently, although the 30 m resolution may not be able to meet all the needs in some details, it still provides an overall understanding of the vegetation condition in the Shendong Coal Base and serves as a valuable reference and data support for assessing the loss of vegetation carbon sinks in the region. As remote sensing and methods for calculating VCS continue to evolve, future studies will increasingly rely on higher resolution VCS data. This allows for the capture of more subtle vegetation characteristics and changes, as well as the assessment of the dynamics of vegetation carbon sinks in mining areas.

### Impact of coal mining on VCS

Currently, the development of China's coal resources has shifted towards the western region, which has a fragile ecological environment. This development is mainly concentrated in four provinces: Shanxi, Shaanxi, Mongolia, and Xinjiang. The Shendong coal base is situated at the intersection of Shanxi, Shaanxi, and Mongolia and comprises 37% of China’s open-pit mines. The conflict between protecting the ecological environment and exploiting resources in these regions is a prominent issue that has attracted the attention of scholars. In 2020, China committed to the United Nations to “strive for peak carbon dioxide emissions by 2030 and carbon neutrality by 2060”. Implementing this strategy has led to active ecological protection and restoration projects. The quantification of the loss of VCS in the mining areas and its changing pattern is of utmost urgency^48^. In a previous study, the author reported that as of 2021, 400.08 km^2^ of vegetation in the Shendong coal base had been destroyed, while 177.91 km^2^ had been restored. However, the changes in VCS during coal mining and restoration have not been quantified. This paper explores the impact of coal production on VCS from two perspectives, providing a new analytical approach for scientifically assessing the impact of coal development activities on VCS.

The process of vegetation change, including both the transition from presence to absence and from absence to presence, can be easily observed. Therefore, measuring the changes in VCS from one year to the next is common practice, referred to as the direct changes in VCS. This measure is frequently employed by coal companies to evaluate the effectiveness of restoration efforts. The study found that the increase and decrease in VCS were directly associated with trends in vegetation destruction and restoration in the study area. Consequently, the direct change in VCS most directly reflects the intensity of mining and restoration activities carried out each year, as well as the magnitude of the impact on VCS. The fluctuating relationship shown in Fig. 2b provides crucial information on the effectiveness of restoration activities. By analyzing the fluctuating relationship between C_diper_ and C_ddper_, it is possible to assess the effectiveness of restoration in different years. This is crucial for the development of more effective vegetation restoration strategies and the improvement of the efficiency of ecological restoration. Overall, the R_VCS_ and R_V_ were almost equal. However, only in some years (e.g., 2003, 2004, and 2016), the R_VCS_ exceeded the R_V_. Combining with the trend of VCS in the mining area, it is evident that VCS had a stage of high value in these years (see Fig. 1a). Restoration activities that are tailored to natural conditions can enhance restoration efficiency. It is worth noting that the R_VCS_ has consistently lagged the R_V_ since 2017, with a significant increase in the disparity between the two after 2019. It can be inferred that although the vegetation area was effectively restored from 2017 to 2022, its VCS capacity was not fully restored. This is further supported by the fact that C_restore remained below 0 after 2019, as shown in Fig. 2c, indicating that VCS did not return to its original level. This discrepancy may be attributed to the restoration work mainly focusing on planting and covering vegetation while neglecting the integral role of carbon fixation during vegetation growth and development. In order to ensure successful restoration efforts in mining areas, it is crucial to not only prioritize the restoration of vegetation cover, but also to focus on restoring ecosystem functionality, including the restoration of carbon sequestration capacity.

The impact of mining activities on the annual VCS was assessed by comparing it with its original state. The potential changes in VCS followed an “S” curve increase due to the accumulation of VCS losses over time (refer to Fig. 2d). This feature is related to the development stages of China’s coal industry. From 2001 to 2011, the coal mining industry experienced rapid growth, resulting in the destruction of a substantial amount of vegetation and a significant loss of VCS. However, between 2012 and 2017, the industry underwent a phase of layout optimization, leading to a progressive decrease in coal production. During this period, the increase in VCS losses was effectively mitigated. After 2017, there was a resurgence in mining intensity, leading to a subsequent increase in the loss of VCS. Spatially, the changes in VCS resulting from unit restoration areas showed a spatial distribution trend with the southwest-northeast direction as the dividing line (see Fig. 3b). The VCS of mines located in the northwest direction were able to restore to the original level after the restoration activities, with a positive value of C_restore indicating restoration success. However, the VCS of the mines in the southeast direction was not fully restored to its original state. One possible explanation for this difference is the regional distribution of NDVI (see Supplementary Fig. S1). The NDVI was significantly lower in the northwest region, indicating a less pristine vegetation status than in the southeast region. As a result, restoration efforts in the northwest region are more likely to exceed the pristine status of the vegetation. Consequently, the spatial distribution trends observed in Fig. 3b accurately reflect the extent of the impact of restoration activities on potential changes in VCS at different mine sites. This further emphasizes the critical role of the potential changes in VCS per unit of restoration area in reflecting the effectiveness of restoration activities. In a coal company, restoration completion is typically considered as compensation for direct losses to VCS caused by mining. However, it is essential to note that losses in destroyed areas will continue to accumulate over the life of the mine compared to the original state of vegetation. Therefore, compensating for the loss of VCS in the year of destruction does not fully restore the ecological benefits. The paper aims to raise awareness of the need for vegetation restoration and carbon sequestration compensation by calculating the potential ecological impacts of coal development activities. This information can serve as a valuable reference for developing and implementing policies related to coal mining and ecological restoration.

## Materials and Methods

### Study area and data

The Shendong coal base is located at the junction of Inner Mongolia, Shaanxi, and Shanxi in China. It belongs to the Yellow River Basin and has geographical coordinates ranging from 38°42′-40°06′N and 109°41′E—111°36′E (see Supplementary Fig. S1). The Shendong coal base is one of China’s 14 major coal bases, with a total area of 18,393.7 km^2^ and proven coal reserves of 223.6 billion tonnes. This region has an ecologically fragile environment and a semi-arid continental monsoon climate, with natural and restored vegetation is dominated by grasslands and sparse shrubs. The region receives an average annual precipitation of approximately 386.82 mm, while the average annual temperature is around 7.36 °C. Since 2007, the Shendong coal base has undergone large-scale mining, resulting in the loss of 400.08 km^2^ of vegetation as of 2021^49^. This has significantly impacted the local ecological environment.

The spatio-temporal data on vegetation disturbance from 2001 to 2021 were sourced from a previous study conducted by the authors at the Shendong coal base using an automatic method (Auto-VDR) for identifying vegetation destruction and restoration of various open-pit mines^45^. Images from 2012 were excluded because of the poor data quality. The data had a spatial resolution of 30 m. To ensure more accurate data for analysis, we manually inspected the recognition errors based on remotely sensed imagery. For the missed and misidentified regions shown in Supplementary Fig. S2, we reviewed all Landsat and GF imageries in turn for the years 2002–2022. The initial data were corrected because surface mining can cause significant changes in feature types, and the destruction time and restoration time were easily identifiable in the imagery. Based on this, the destruction and restoration areas for 2022 have been added. The accuracies for vegetation destruction time and restoration time were 0.94 and 0.92 after pre-processing, respectively (see Supplementary Fig. S3-4 online).

The annual net primary productivity (NPP) of vegetation in the Shendong coal base from 2001 to 2022 was calculated using the global MODIS NPP product MOD17A3HGF v061 with 500 m spatial resolution, which was acquired from the National Aeronautics and Space Administration (NASA) (https://lpdaac.usgs.gov/). This NPP product was estimated using the BIOME-BGC (BioGeochemical Cycles) model^50^. The annual NPP is derived from the sum of all 8-day Net Photosynthesis (PSN) products (MOD17A2H) from the given year^51^.

### Scale transformation of NPP

**Figure 4:**
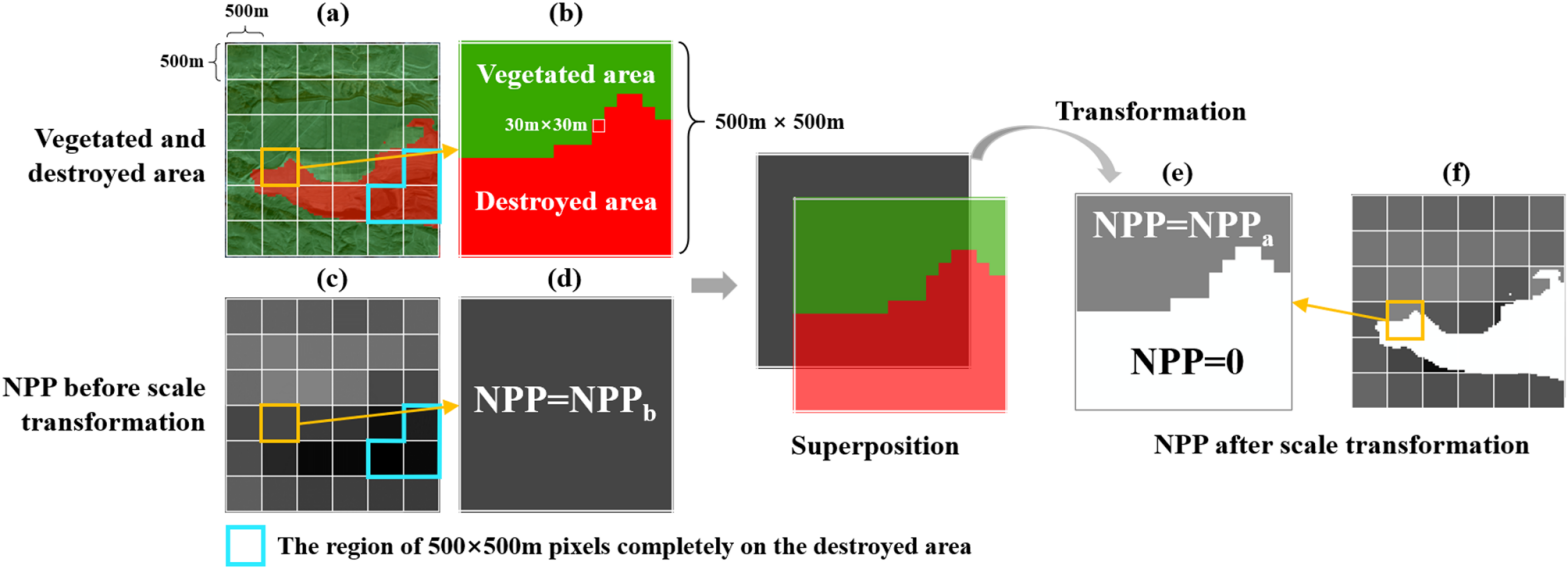
Scale transformation of NPP data. (**a**) The color red indicates areas where destruction has occurred and green indicates areas of vegetation. The blue circle highlights the region of 500 × 500 m pixels completely on the destroyed area. (**b**) An enlarged 500 × 500 m grid with vegetation and damaged area data at 30 m resolution. (**c**) NPP data at 500 m resolution. (**d**) An enlarged 500 m grid with NPP as NPP_b_. (**e**) Assign a value of 0 to the destroyed area and calculate the NPP of vegetated area using Eq. (1). (**f**) NPP after scale transformation.

The spatio-temporal data on vegetation disturbance were obtained based on the maximum NDVI data during the growing season (July–September). Therefore, the area identified as destroyed does not necessarily indicate that it was utterly devoid of vegetation throughout the entire year. Instead, it was detected as devoid of vegetation during the growing season. Vegetation may have been present in the destroyed area, accumulating fixed carbon in the months before its destruction, resulting in a lower annual NPP value known as the background NPP (NPP_bg_). The spatial resolution of the NPP data used in this study is 500 m, while the spatio-temporal data on vegetation disturbance has a resolution of 30 m. In some cases, vegetated and destroyed areas may exist within a 500 × 500 m area, as shown in Fig. 4a,b. Therefore, the NPP of this pixel comprises both the NPP of vegetation and the NPP_bg_ of the destroyed area. Before conducting statistical analyses, it is necessary to scale transformation of the existing NPP to obtain the NPP of the vegetated area at a resolution of 30 m. The processing is illustrated in Fig. 4.

(i) To estimate NPP_bg_ in the destroyed area, we randomly selected the 500 × 500 m pixels wholly destroyed within the study area (the blue area in Fig. 4c) and set their NPP as NPP_bg_. The background values of the destroyed area for the entire study area were obtained through Kriging spatial interpolation. These NPP_bg_ are calculated annually.

(ii) To calculate the NPP in the vegetated area, the total area (S) and the vegetated area (S_veg_) for the 500 × 500 m region were calculated separately. Subsequently, the total NPP in this region was calculated as NPP_b_ × S. To obtain the NPP of the vegetated area at a resolution of 30 m, we subtracted the contribution of NPP_bg_ from the fixed total NPP. The resulting NPP was then evenly distributed over the vegetated area based on its area, as shown in Fig. 4e. A value of 0 was assigned to the NPP of the destroyed area. Equation (1) shows the calculation for scale transformation. The NPP after the scale transformation is shown in Fig. 4f.

1 $$ NPP_{a} = \left\{ {\begin{array}{*{20}c} {\frac{{NPP_{b} \times S - NPP_{bg} \times (S - S_{veg} )}}{{S_{veg} }}{\text{, pixel is vegetation }}} \\ { \, 0{\text{ , pixel is not vegetation}}} \\ \end{array} } \right. $$

where, *NPP*_*a*_ represents the NPP after scale transformation (g C m^-2^ a^-1^), while *NPP*_*b*_ represents the NPP before scale transformation (Fig. 4d). *S—S*_*veg*_ refers to the area of destroyed area (m^2^).

### Calculation of VCS

Research has demonstrated that 1 g of carbon in vegetation equals 2.2 g of organic matter. Based on the chemical equation for photosynthesis, vegetation absorbs 1.63 g of CO_2_ for every gram of accumulated organic matter^52^. This conversion relationship can transform NPP into VCS, as shown in Eq. (2).

2 $$ VCS = NPP \times 2.2 \times 1.63 $$

where VCS represents the amount of CO_2_ fixed by vegetation per unit area and time, which is represented by carbon sequestration in vegetation (VCS) in this paper (unit: g CO_2_ m^-2^ a^-1^). The coefficient of conversion from NPP to organic matter is 2.2, and the coefficient of conversion from organic matter to CO_2_ is 1.63.

### Calculation of VCS of undisturbed state in the mining areas

A linear regression model was fitted using the pre-mining (2001-T_D_) VCS data of the study area, as shown in Eq. (3). The VCS of undisturbed state after the destruction time was then predicted based on the regression equation, as shown in Eq. (4), and the results formed a “Prediction line”. The predicted VCS of undisturbed state represent the original state of the VCS when the study area is assumed to be unaffected by mining activities.

3 $$ a = \frac{{n\sum {tVCS_{t} - \sum t } \sum {VCS_{t} } }}{{n\sum {t^{2} } - (\sum t )^{2} }}, \, b = \frac{{\sum {VCS_{t} } }}{n} - a\frac{\sum t }{n}, \, t = 2001, \cdots ,T_{D} $$

4 $$ VCS_{Year} = a \cdot Year + b, \, Year = T_{D} + 1, \cdots ,2022 $$

where *n* represents the total years involved in the regression, *T*_*D*_ denotes the vegetation destruction time, *t* is the year, and *VCS*_*t*_ denotes the VCS for the respective year.

### Quantification of direct and potential changes in VCS

This paper analyzes the changes in VCS in the Shendong coal base from two perspectives (see Supplementary Fig. S4 online). The coal development activities have caused destruction and restoration of vegetation, resulting in changes in VCS. The term “direct changes in VCS” refers to changes relative to the previous year when the destruction or restoration occurred (refer to Fig. S4a), including direct decrease and direct increase. “Potential changes in the VCS”, on the other hand, are relative to the undisturbed state (Fig. S4b). To distinguish potential changes in VCS caused by mining and restoration activities, we labeled the potential changes in destroyed area as C_destroy and in restored area as C_restore. The restoration rate of vegetation (R_V_) is defined as the ratio of the restoration area over the destruction area, while the restoration rate of VCS (R_VCS_) is defined as the ratio of “the direct increase in VCS” over “the direct decrease in VCS”. All abbreviations used in this paper are summarized in Supplementary Table S2 online.

The deficit of carbon sequestration in vegetation (VCSD) from surface coal development activities was calculated using Eq. (5). VCSD is defined as the total reduction in VCS compared to the undisturbed state at the mine sites after vegetation destruction has occurred.

5 $$ VCSD = - (\sum\nolimits_{{{\text{t}} = 2001}}^{2022} {C\_destroy_{t} } + \sum\nolimits_{{{\text{t}} = 2001}}^{2022} {C\_restore_{t} } ) $$

where *VCSD* is the deficit of carbon sequestration in vegetation, *C_destroy*_*t*_ is the potential changes in VCS in the destroyed area in year *t*, and *C_restore*_*t*_ is the potential changes in VCS in the restored area in year *t*. If *VCSD* is greater than 0, it indicates that coal development activities have had a negative impact on VCS. Conversely, if *VCSD* is less than 0, it indicates that coal development activities have increased VCS in the mining area.

### Supplementary Information

Supplementary Information.

## Data Availability

The datasets generated during and/or analyzed during the current study are available from the corresponding author on reasonable request. The MOD17A3HGF NPP dataset is from https://lpdaac.usgs.gov/products/mod17a3hgfv061/.
