# Supplementary material for: Dynamics of carbon sequestration in vegetation affected by large-scale surface coal mining and subsequent restoration
Source: Sci Rep. 2024 Jun 12;14:13479. doi: 10.1038/s41598-024-64381-1 (PMC11169282; doi:10.1038/s41598-024-64381-1)
Supplement: Supplementary file 1 — Supplementary Information. [file 41598_2024_64381_MOESM1_ESM.docx]

Supplementary Information for

**Dynamics of Carbon Sequestration in Vegetation Affected by Large-Scale Surface Coal Mining and Subsequent Restoration**

Yaling Xu *et al.*

*Corresponding author. Email: Jun Li, [junli@cumtb.edu.cn](mailto:junli@cumtb.edu.cn)

**This PDF file includes:**

Figs. S1 to S5

Tables S1 to S2

Fig. S1. The location of the study area. The Shendong coal base is located at the junction of Inner Mongolia, Shaanxi, and Shanxi in China. The natural vegetation in the area is displayed in the top right corner, featuring both camera and drone shots. The topographic data in the top left corner is derived from GEBCO Compilation Group (2023) GEBCO 2023 Grid (doi:10.5285/f98b053b-0cbc-6c23-e053-6c86abc0af7b).


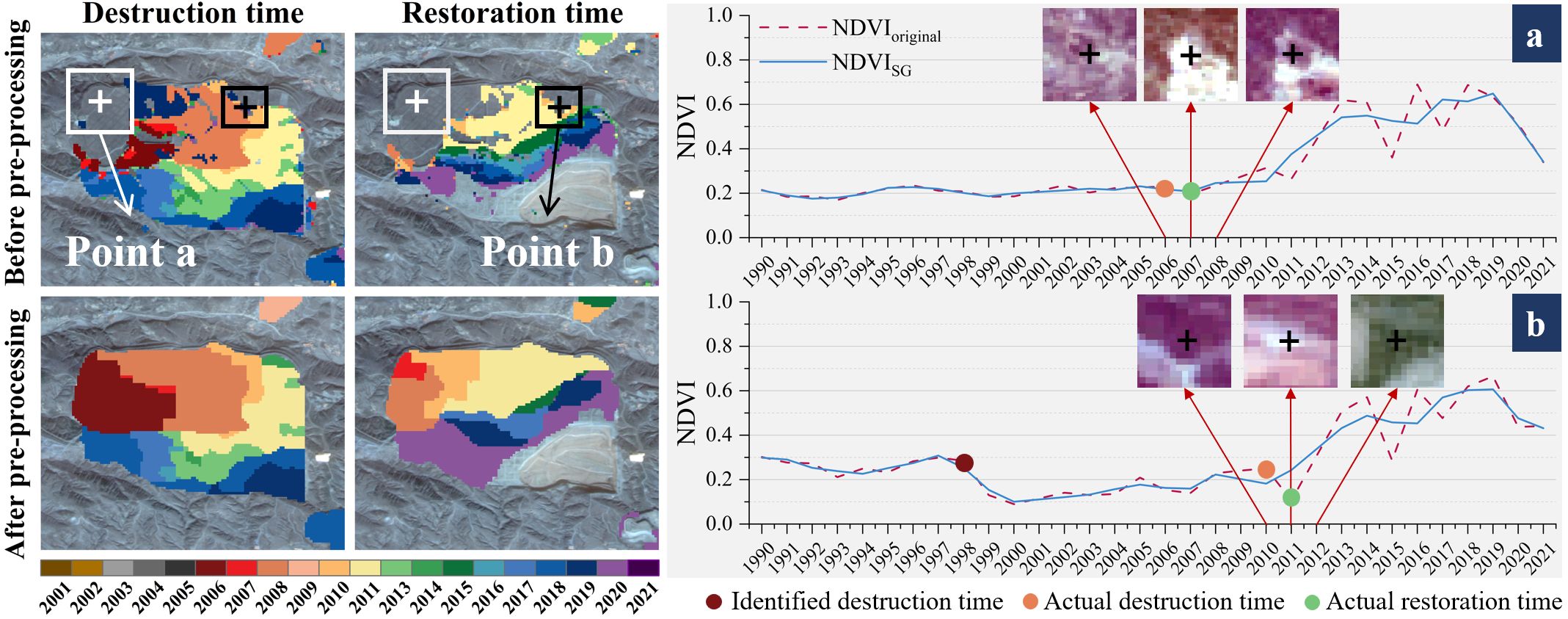


Fig. S2. Pre-processing of vegetation destruction and restoration data. Curve a corresponds to point a, which represents destroyed areas that were incorrectly classified as undestroyed; Curve b corresponds to point b, which represents misidentification due to poorly characterized NDVI curves





Fig. S3. Accuracy of destruction time and restoration time after pre-processing. The accuracy for destruction time increased from 0.82 to 0.94, while the accuracy for restoration time increased from 0.87 to 0.92. The user and producer accuracies for the destruction time exceeded 0.9. Similarly, the user and producer accuracies for restoration time exceeded 0.85, except for 2010 and 2016, which still met the requirements for subsequent analyses.


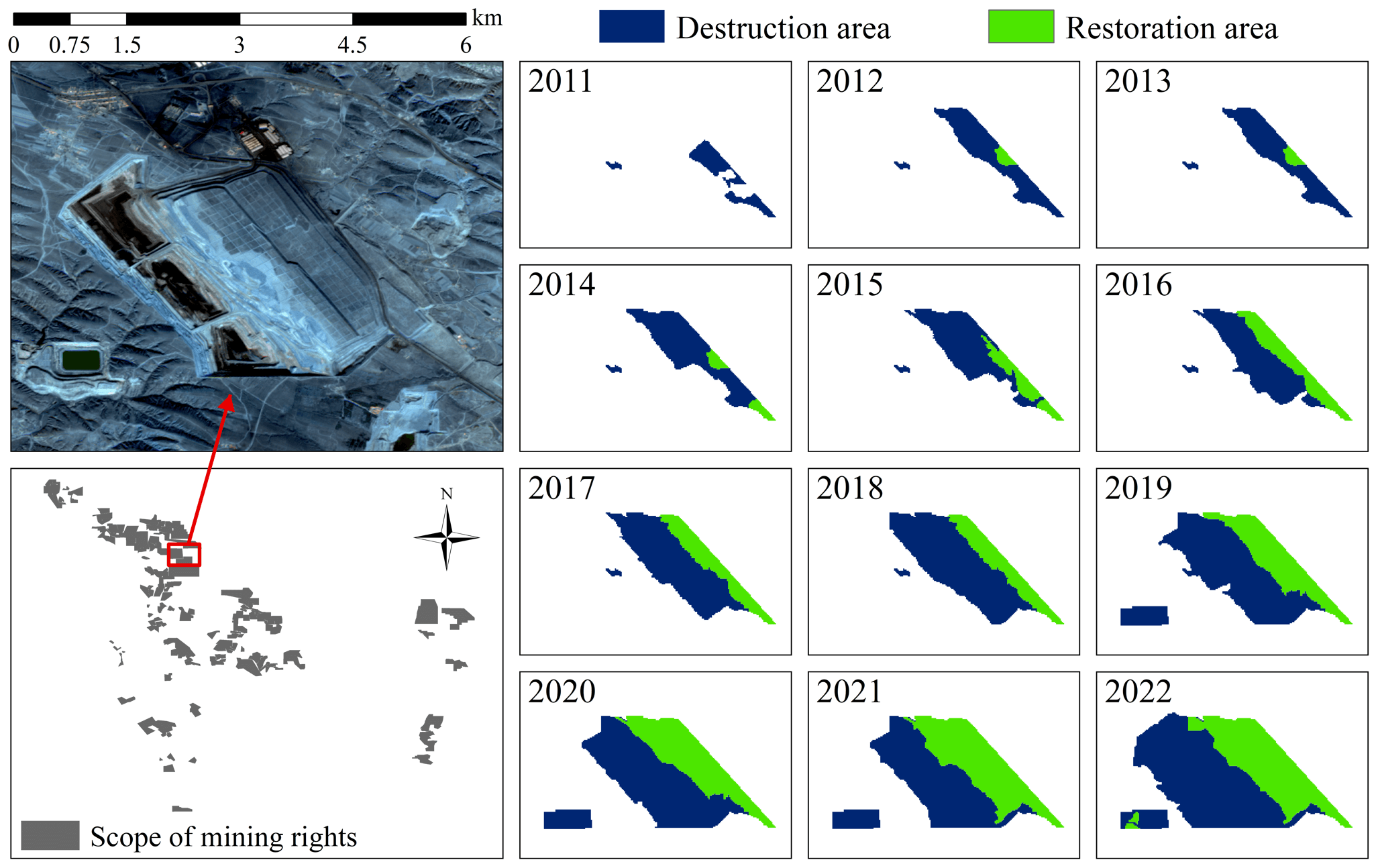


Fig. S4. The regions of vegetation destruction and restoration, with a typical mine as an example.


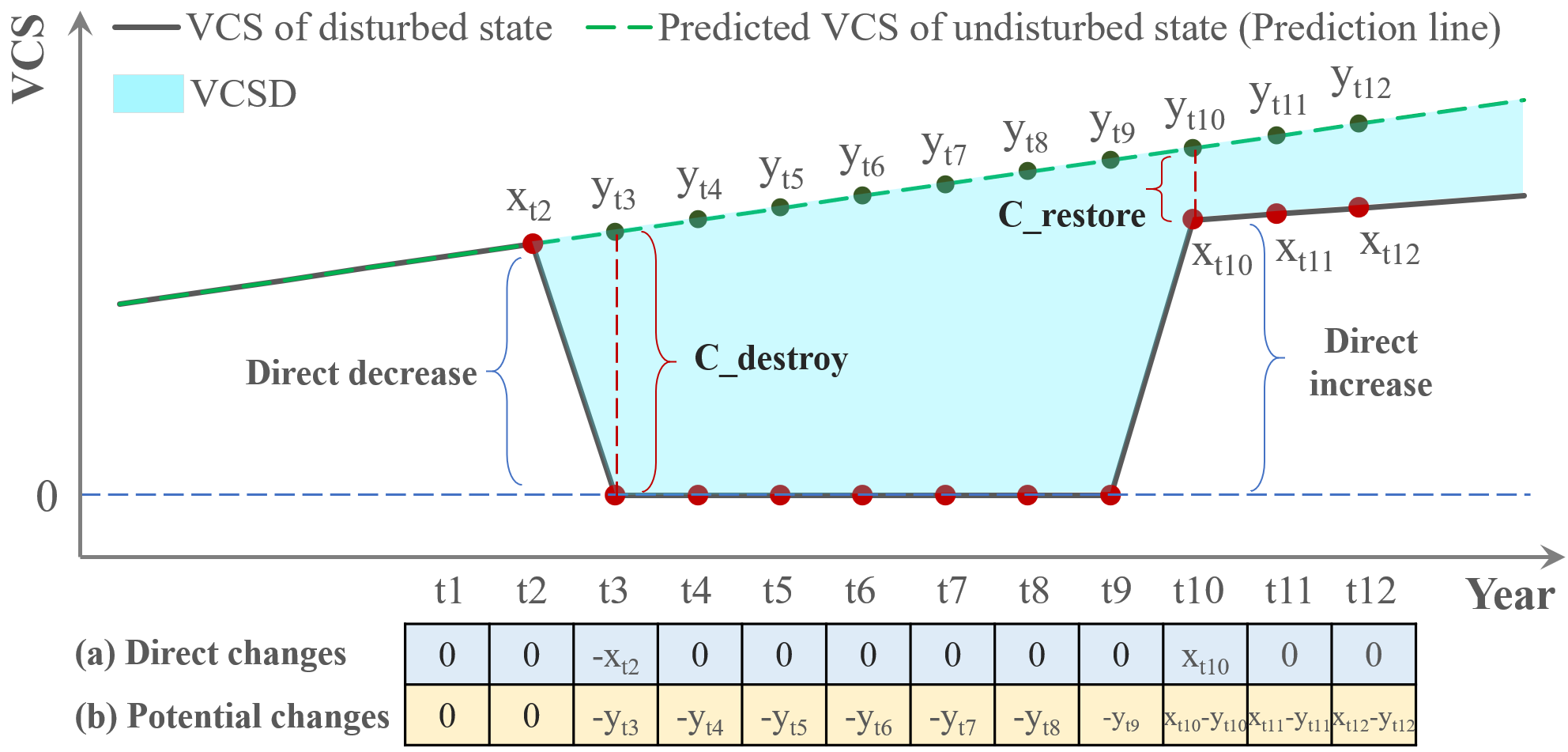


Fig. S5 Modelling for quantification of changes in VCS. Prediction line is the regression equation derived from the modeling in Equation (4). For example, mining occurred at time t4, resulting in a change in VCS of the destroyed area from x_t2_ in the previous year to 0, with a direct change in VCS of -x_t2_ (where “-” indicates a decrease, i.e., the direct decrease in VCS is x_t2_). Between t4 and t9, no activities related to destruction or restoration occurred, so no direct changes in VCS registered. Similarly, restoration occurred at time t10, causing the VCS of the restored area to change from 0 in the previous year to x_t10_, resulting in a direct change in VCS of x_t10_ (where “+” indicates an increase, i.e., the direct increase in VCS is x_t10_). Following the mining activities at time t4, the VCS remains at 0 throughout the mining process, resulting in the loss of its initial value each year. An example of calculating potential changes in VCS is as follows. For example, at time t4, the VCS of y_t4_ is still lost, leading to a potential change in the VCS of -y_t4_. After a restoration activity at time t10, if x_t10_ is greater than y_t10_, it is considered that the ecosystem has increased its VCS by x_t10_ - y_t10_. If x_t11_ is less than y_t11_, the area is still considered to have decreased its VCS by y_t10_ - x_t10_.

Table S1.

The annual R_V_ and R_VCS_ in the study area.

| **Year** | **Destruction** | | **Restoration** | | **R_V_** | **R_VCS_** |
| --- | --- | --- | --- | --- | --- | --- |
|  | **Area**  **(km^2^)** | **Direct decrease**  **(Gg CO_2_)** | **Area**  **(km^2^)** | **Direct increase**  **(Gg CO_2_)** |  |  |
| 2001 | 0.15 | 0.08 | 0.11 | 0.05 | 0.73 | 0.58 |
| 2002 | 0.11 | 0.04 | 0.00 | 0.00 | 0.00 | 0.00 |
| 2003 | 0.24 | 0.11 | 0.02 | 0.01 | 0.09 | 0.16 |
| 2004 | 0.41 | 0.21 | 0.01 | 0.01 | 0.02 | 0.03 |
| 2005 | 1.32 | 0.74 | 0.05 | 0.03 | 0.04 | 0.04 |
| 2006 | 4.35 | 2.51 | 0.04 | 0.02 | 0.01 | 0.01 |
| 2007 | 5.24 | 3.14 | 0.13 | 0.07 | 0.02 | 0.02 |
| 2008 | 15.89 | 10.32 | 0.57 | 0.39 | 0.04 | 0.04 |
| 2009 | 61.11 | 41.85 | 1.33 | 0.72 | 0.02 | 0.02 |
| 2010 | 13.95 | 9.84 | 0.14 | 0.12 | 0.01 | 0.01 |
| 2011 | 44.25 | 31.60 | 3.26 | 2.07 | 0.07 | 0.07 |
| 2013 | 62.79 | 48.51 | 46.67 | 33.93 | 0.74 | 0.70 |
| 2014 | 24.08 | 20.90 | 19.77 | 17.81 | 0.82 | 0.85 |
| 2015 | 18.77 | 15.96 | 10.26 | 7.99 | 0.55 | 0.50 |
| 2016 | 12.67 | 11.03 | 25.89 | 27.14 | 2.04 | 2.46 |
| 2017 | 25.61 | 22.55 | 11.90 | 9.86 | 0.46 | 0.44 |
| 2018 | 38.25 | 36.35 | 19.07 | 16.72 | 0.50 | 0.46 |
| 2019 | 44.86 | 44.58 | 25.60 | 22.79 | 0.57 | 0.51 |
| 2020 | 22.66 | 24.06 | 27.59 | 21.76 | 1.22 | 0.90 |
| 2021 | 17.80 | 19.00 | 21.47 | 18.67 | 1.21 | 0.98 |
| 2022 | 38.33 | 41.23 | 13.62 | 12.34 | 0.36 | 0.30 |
| **Total** | **452.85** | **384.63** | **227.52** | **192.51** | **0.50** | **0.50** |

Table S2.

Abbreviations used in this article.

| Abb | Full name | Abb | Full name |
| --- | --- | --- | --- |
| VCS | Carbon Sequestration in Vegetation | R_V_ | The restoration rate of vegetation |
| NDVI | Normalized Difference Vegetation Index | R_VCS_ | The restoration rate of VCS |
| NPP | Net Primary Productivity | NPP_bg_ | The background NPP value |
| C_diper_ | the direct decrease in VCS per unit of restored area | | |
| C_ddper_ | the direct increase in VCS per unit of destroyed area | | |
| C_destroy | Potential changes in VCS in the destroyed area | | |
| C_restore | Potential changes in VCS in the restored area | | |
| VCSD | The deficit of carbon sequestration in vegetation | | |
